# Open fractures and the incidence of infection in the surgical debridement 6 hours after trauma

**DOI:** 10.1590/1413-78522015230100932

**Published:** 2015

**Authors:** Miguel de Castro Fernandes, Luciano Rodrigo Peres, Aristóteles Correia de Queiroz, José Queiroz Lima, Flávio Moral Turíbio, Marcelo Hide Matsumoto

**Affiliations:** Casa de Saúde Santa Marcelina, São Paulo, SP, Brazil, Casa de Saúde Santa Marcelina (CSSM), São Paulo, SP, Brazil

**Keywords:** Fractures, open, Infection, Debridement

## Abstract

**Objectives:**

To determine whether a time delay greater than 6h from injury to surgical debridement influences the infection rate in open fractures.

**Methods:**

During a period of 18 months, from October 2010 to March 2012, 151 open fractures were available for study in 142 patients in our hospital. The data were collected prospectively and the patients were followed up for 6 weeks. The patients were divided into two groups regarding the time delay from injury to surgical debridement (more or less than 6 hours).

**Results:**

Surgical debridement was carried out in less than 6h from injury in 90 (59.6%) fractures and after 6 hours from injury in 61 (40.4%) fractures. Infection rates were 12.22% and 13.24%, respectively. The global infection rate was 13.24%.

**Conclusion:**

A significantly increased infection rate was not observed in patients whose surgical debridement occurred more than 6h after injury. However, in the fractures of high-energy trauma, a statistically significant increase of the rate of infection was observed in those operated 6 hours after trauma. Level of Evidence II, Study Type Comparative and Prospective.

## INTRODUCTION

Treatment of open fractures consists of immobilization, antibiotics, tetanus prophylaxis, surgical wound debridement, reduction and fixation of the fracture and restoration of soft tissue coverage,^1^ the outcome being associated with the severity of the initial trauma and these variables.^2^^-^ 3

The protocols that establish the best time for surgical debridement of open fractures within 6 hours early trauma, in order to reduce the risk of deep infection, emerged from the classic work of Friedrich^4^ (1898). This recommendation was established before the onset of the basic protocol of life support in 1960^5^^,^ 6 and advanced life support in 1978,^7^^-^ 9 of modern antibiotics in 1932,^10^ of pulsatile lavage and systematic debridement. The time elapsed between trauma and surgical debridement is sometimes greater than 6 hours, resulting of a variety of factors, including the need for treatment of associated injuries before the surgical treatment of fractures, delayed transfer of patients from other health units and logistical issues, such as the availability of operating room. Recent studies have used this fact to assess whether the delay in surgery changes the treatment outcome.^2^^,^ 11 ^-^ 23

Over a period of 18 months, from October 2010 to March 2012 142 patients with 151 fractures who underwent surgical debridement and cleaning according to the service protocol were prospectively assessed.

The objective was to compare the outcome of open fractures addressed before and after 6 hours of injury with regard to the development of infection in a 6 weeks period.

## MATERIALS AND METHODS

This is a not randomized comparative study in which the patients studied were didactically divided according to the time elapsed from the moment of trauma to the surgical debridement; one of the groups included those individuals undergoing the procedure within 6 hours following the trauma and the other those whose debridement was performed 6 hours after the event. The study was approved by the Ethics Research Committee of the hospital under protocol number 51/2010. All patients authorized their participation in the study through the Informed Consent form.

To set the time taken from the moment of trauma to the surgical procedure the following sources were used: the patient's and/or family own information, the record of the rescue service (time of trauma) and the record of the beginning of orthopedic surgery in the medical record of each patient (time of surgery).

The study included patients admitted for convenience (on demand and/or transfer) at the orthopedic emergency room, from October 2010 to March 2012, of various kinds of trauma victims, with open fractures, treated according to the service protocol:

1. The wounds were clinically evaluated, subjected to surface cleaning and placement of sterile dressing;
2. Temporary stabilization of the fracture;
3. Antibiotic therapy was performed at admission and during hospitalization according to the CCIH protocol;
4. Anti tetanus prophylaxis.

Debridement, surgical cleaning with saline and surgical stabilization of the fracture were performed. Progression to infection in the first 6 weeks after injury was then evaluated through clinical criteria (redness, warmth, swelling or presence of secretion with infectious aspect) and/ or complementary tests (Leukogram, ESR, PCR, cultures). The following data were collected through interviews with the patient and data from medical records: time of the accident; accident location; mechanism of injury; site of the first aid; time elapsed between the time of trauma and emergency surgery; type of surgical procedure performed at the initial treatment; type of definitive surgical procedure; time elapsed between the time of trauma and the onset of antibiotic therapy; length of hospital stay; fracture topography (affected bone); fracture classification (OA/OTA, Gustilo-Anderson, Tscherne); presence of associated injuries on admission; non orthopedic surgical procedure previous to debridement; presence of infection in 6 weeks after trauma. 

Statistical analyzes were performed using the SPSS V16 software, Minitab 15 and Excel Office 2010. In order to assess the homogeneity between categories of qualitative variables and evaluate the association between variables the tests ANOVA, chi-square and odds ratio were used.

The study has a sample size exceeding 20 subjects, which, according to the Central Limit Theorem, it ensures that the data tends to a normal distribution. In addition, the normality of residues of this statistical model was tested (Kolmogorov-Smirnov normality test) and was found to be normal, which allows the use of ANOVA.

For this study a 0.05 (5%) significance level was defined and all confidence intervals built along the study were made with 95% statistical confidence.

The study included patients admitted for convenience (free demand and/or transfer) at the orthopedic emergency room of the Unified Health care System (SUS), between October 2010 and March 2012, with various kinds of trauma victims, presenting compound fractures and treated according to the service protocol. We excluded patients with compound fractures of the hand, distal to the wrist, foot, distal to tarsal-metatarsal joint and those involving the bones of the face and skull, because they present a different infection rate than long bones,24 and patients submitted to amputation as emergency treatment due to member unviability and those whose follow up was not possible (transfer, death).

## RESULTS

During the time of study, 153 patients were admitted into the hospital with 162 compound fractures. One patient died as a result of trauma, six were admitted receiving indication of immediate amputation and four did not return in consultation for unknown reasons. These patients were excluded from the study.

Thus, 142 patients remained in the study with 151 open fractures. The mean age of patients was 31.76 (range 3-87) years old. Of these, 118 (78.14%) were male and 33 (21.85%) females.

**Figure 1.**
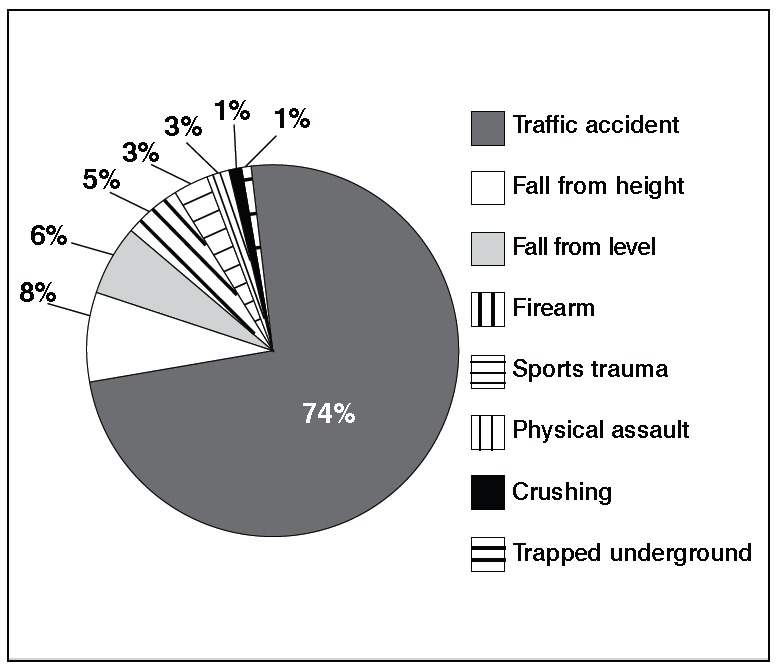
Distribution of compound fractures according to trauma mechanism.

**Figure 2.**
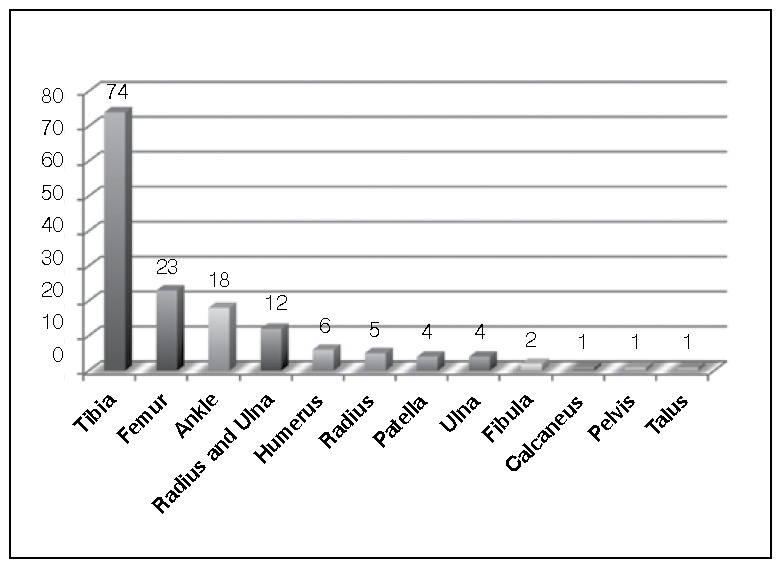
Distribution of compound fractures according to anatomic location.

Traffic accidents predominated as the main trauma mechanism, involving 112 (74.18%) patients. Other trauma mechanisms were fall from height (7.95%), fall from level (6.63%), injuries by firearms (4.64%), sports trauma (2.64%), physical aggression (1.98%), crushing (1.32%) and underground trapping (0.66%). In the study we observed 76 tibia fractures, which is the most affected bone, corresponding to 50.3% of the fractures. (Figures 1 and 2)

**Figure 3.**
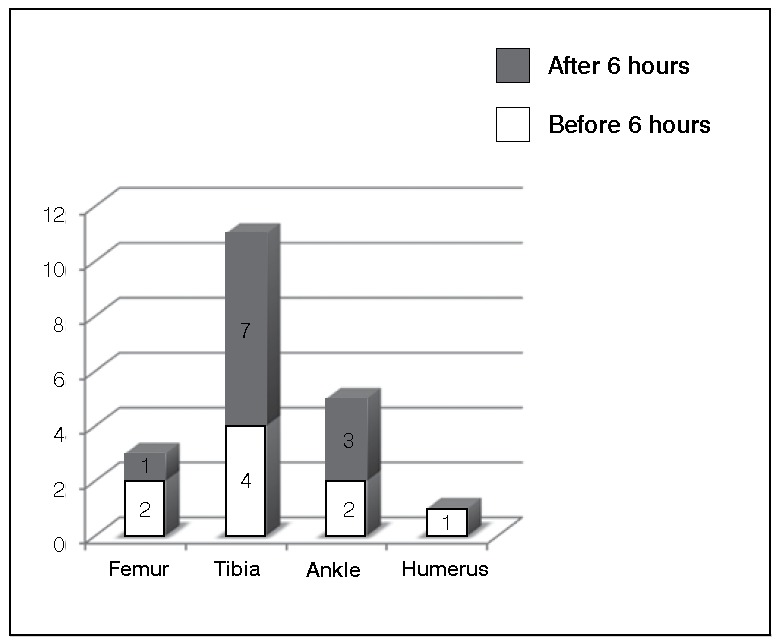
Distribution of infected compound fractures according to anatomical location.

There were 20 (13.24%) cases of infection overall, 11 (55%) affecting leg bones. Of these, 7 (35%) were in the group operated within the first 6h. It drew our attention the high incidence of infection in ankle fractures, with five cases in 18 fractures (27.78%). (Figure 3)

The methods used to stabilize the fractures in the emergency room were isolated external fixator (69.29%), external fixator associated with minimal internal synthesis (3.15%), plate and screw (6.3%), intramedullary rod (0, 79%), Kirschner wires (2.36%), tension band (3.15%), immobilization or traction splint (14.96%). Of the 151 fractures in the study, 83 (54.96%) of them were subjected to a second surgical procedure to definitive treatment. (Table 1)

**Table 1.** Stabilization method used to treat compound fractures.

Stabilization method	n	Percentage
External fixation	104	68.87
Cast	23	15.23
Plate and screw	9	5.96
External fixation + Kirchner wire	4	2.65
Tension band	4	2.65
Kirchner wire	3	1.99
Skeletal traction	2	1.32
Intramedullary rod	1	0.66
Amputation	1	0.66
Total	151	100.00

**Figure 4.**
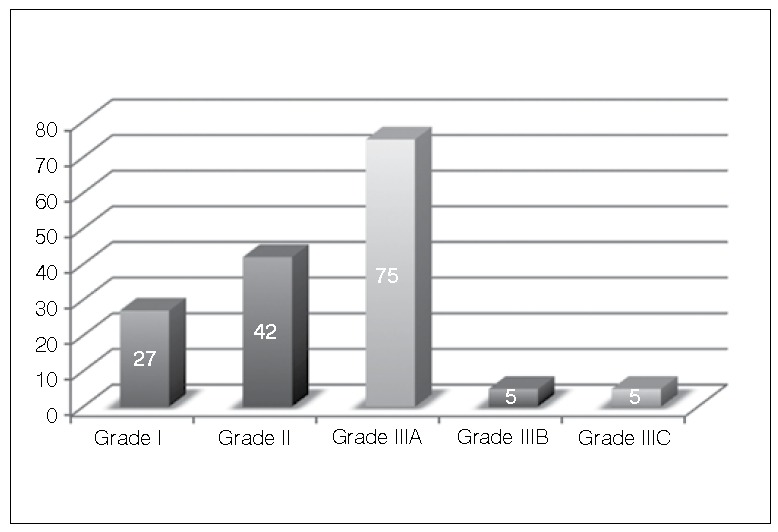
Distribution of compound fractures according to Gustilo classification.

Distributing fractures according to Gustilo and Anderson, 27 fractures grade I (17.88%), 42 grade II (27.81%), and 82 grade III (54.30%) were observed. Of these, 75 were IIIA (49.66 %) 2 IIIB (1.32%), and 5 IIIC (3.31%). (Figure 4)

Among the 20 (13.24%) fractures in which infection was detected, one (5%) fracture was grade I, three (15%) were grade II and 16 (80%) grade III. Analyzing the proportion of infected fractures between grades 1 and 2 (20%) and grade 3 fractures (80%) using the chi-square test, there was a statistically significant difference in fractures addressed after over 6 hours of injury and fractures in general (p=0.001 and p=0.008, respectively). Table 2 shows the distribution of fractures regarding to the classification system of compound fractures of Gustilo and Anderson, ranked by the incidence of infection and time to debridement.

**Table 2.** Relationship between compound fractures according to Gustilo and Anderson classification and time from trauma to surgery, correlated with occurrence of infection.

Gustilo and Anderson classification		Infection						Yes
		No		Yes		Total		
		N	%	N	%	N	%	
≤6 h	Type 1/2	37	47%	4	36%	41	46%	0,514
	Type 3	42	53%	7	64%	49	54%	
>6 h	Type 1/2	31	60%	0	0%	31	51%	0,001
	Type 3	21	40%	9	100%	30	49%	
Geral	Type 1/2	68	52%	4	20%	72	48%	0,008
	Type 3	63	48%	16	80%	79	52%	

Hours (h), greater that (>), lower than or equal to (≤).

The time, in hours, for administration of IV antibiotics (intravenous) from the time of injury was on average 3.05 h (range 1 to 15 h). No patient was administered IV antibiotic by the rescue team. There was no association between time to the administration of IV antibiotic and the incidence of infection in compound fractures studied using ANOVA.

The average time between the moment of the trauma and surgical debridement was 6.42 hours varying from 1h to 20 h. Ninety patients (59.6%) underwent emergency surgery before 6h and 61 (40.4%) after 6h of trauma. The overall infection rate of fractures was 13.24%. Among the fractures treated before 6 h the rate was 12.22% and those debrided after 6h of injury was 14.75%. Using ANOVA we found that, although there are differences in the mean infection rates between subjects with and without infection in both variables, they can not be considered statistically significant (p = 0.652). The analysis using chi-square test also showed no statistically significant difference between the results. Calculating the "odds ratio" between the variables we obtained a value of 1.24 which, despite being a low value, we can say that the patients operated on after 6h are 1.24 times more likely to have infection than patients undergoing the procedure in less than 6h after trauma. (Table 3)

**Table 3.** Relationship of time elapsed between trauma and surgery with infection occurrence

Time between trauma and surgical procedure						
Infection	≤6 h		>6 h		Total	
	N	%	N	%	N	%
Yes	11	12%	9	15%	20	13%
No	79	88%	52	85%	131	87%
Total	90	60%	61	40%	151	100%

Hours (h), greater than (>), lower than or equal to (≤); p-value = 0,652 and Odds Ratio = 1,24.

Citation: Fernandes MC, Peres LR, Queiroz Neto AC, Lima Neto JQ, Turibio FM, Matsumoto MH. Open fractures and the incidence of infection in the surgical debridement 6 hours after trauma. Acta Ortop Bras. [online]. 2015;23(1):38-42. Available from URL: http://www.scielo.br/aob.

## DISCUSSION

Treatment of compound fractures has been the subject of controversy. In hospitals treating patients suffering from trauma, there is consensus that the initial treatment of these fractures should ideally be held in a less than 6 h. This theory is based on the work by Friedrich4 who used garden soil and dust as infectious agents to wounds in experimental animals. In his study he demonstrated that the initial phase of bacterial growth in contaminated wounds terminates within 6 to 8 h after inoculation. After this time, debridement would be less effective to control the wound infection. Friedrich then recommended cleaning and circumferential excision of the wound edges within 6h.

For various reasons, not always surgical debridement can be done within the first 6h. In some cases, the procedure is performed by overwhelmed and tired surgeons and anesthetists at inappropriate times.25-27 A waiting time between 6 and 24h for the surgical treatment of compound fractures can allow better preoperative planning of the definitive treatment fractures, better recognition of the severity of associated injuries, and therefore adequate clinical stabilization. In the current literature, there is no scientific evidence reporting that the delay in surgical debridement interferes in the incidence of infection.

Observational studies have shown an association between the incidence of infection and severe fractures, according to Gustilo and Anderson classification 28,29 and those involving leg bones,30 this fact was also observed in this study. It is noteworthy that in less serious fractures (grade I and II) we observed four infections and in those operated after 6 h no infection was observed, showing that the time was not the only determining factor, i.e. the severity of injuries should always be considered. We, therefore, recommend that more severe fractures must be operated as soon as possible.

It drew our attention the high incidence of infection in ankle fractures, where of the 18 fractures, 27.7% developed infection, a large number when compared with fractures of the leg bones, which 16.9% of the 65 fractures infected. This group alone was not researched isolated, but it would take more studies to better assess the cause of this observation.

Few studies have been able to establish a direct relationship between infection and the delayed surgical debridement.11-13 Other similar studies showed no direct relationship between the frequency of infection, time to surgical debridement and IV antibiotic administration.15-21 Despite there is a possibility of a type II error (due to the limited number of cases) in the present study, we found no statistical significance between the time of debridement and the incidence of infection. Of all fractures 40.4% were operated after 6 h of trauma, however, the reasons which led to the delay to surgical debridement were not the subject of this study.

The compound fractures infection rates vary considerably in the literature. In a national study, Muller *et al.* 23 found acute infectious complications in 20.5% of the fractures. Other international studies also showed similar infection rates to those found in our study,18,28 which showed an overall incidence of 13.24%.

We believe that to better assess the link between the time and the occurrence of infection, a multicenter randomized study would be necessary, but ethical principles hinder this type of analysis. We also cannot forget other factors that influence the occurrence of infection such as patient-related factors (smoking, diabetes and other comorbidities), type of fracture (severity and location of the lesion) and type of surgery (surgeon's experience, aggressiveness debridement of devitalized tissues and type of synthesis prescribed).

## CONCLUSION

In the overall evaluation, we found no statistically significant difference in the rate of infection in compound fractures addressed before or after 6h of trauma. However, on the most severe fractures, there was evidence of increased infection rate in those operated after 6h.
